# A Tale of Four “Carp”: Invasion Potential and Ecological Niche Modeling

**DOI:** 10.1371/journal.pone.0005451

**Published:** 2009-05-06

**Authors:** Shannon C. DeVaney, Kristina M. McNyset, Justin B. Williams, A. Townsend Peterson, Edward O. Wiley

**Affiliations:** The University of Kansas, Natural History Museum and Biodiversity Research Center, Lawrence, Kansas, United States of America; Monash University, Australia

## Abstract

**Background:**

Invasive species are a serious problem in ecosystems, but are difficult to eradicate once established. Predictive methods can be key in determining which areas are of concern regarding invasion by such species to prevent establishment [1]. We assessed the geographic potential of four Eurasian cyprinid fishes (common carp, tench, grass carp, black carp) as invaders in North America via ecological niche modeling (ENM). These “carp” represent four stages of invasion of the continent (a long-established invader with a wide distribution, a long-established invader with a limited distribution, a spreading invader whose distribution is expanding, and a newly introduced potential invader that is not yet established), and as such illustrate the progressive reduction of distributional disequilibrium over the history of species' invasions.

**Methodology/Principal Findings:**

We used ENM to estimate the potential distributional area for each species in North America using models based on native range distribution data. Environmental data layers for native and introduced ranges were imported from state, national, and international climate and environmental databases. Models were evaluated using independent validation data on native and invaded areas. We calculated omission error for the independent validation data for each species: all native range tests were highly successful (all omission values <7%); invaded-range predictions were predictive for common and grass carp (omission values 8.8 and 19.8%, respectively). Model omission was high for introduced tench populations (54.7%), but the model correctly identified some areas where the species has been successful; distributional predictions for black carp show that large portions of eastern North America are at risk.

**Conclusions/Significance:**

ENMs predicted potential ranges of carp species accurately even in regions where the species have not been present until recently. ENM can forecast species' potential geographic ranges with reasonable precision and within the short screening time required by proposed U.S. invasive species legislation.

## Introduction

Invasive species [2] pose both ecological [3], [4] and economic [5] risks to native ecosystems. Unfortunately, once established, invasives are generally difficult or impossible to eradicate (consider, for example, the case of northern snakeheads *Channa argus* in Maryland; [6]). Robust methods for anticipating the geographic potential of possible invaders on a continental scale would allow decision-makers and managers to make informed decisions and take effective actions towards excluding harmful species before they are established. Previous investigators have applied ecological niche modeling (ENM) to this problem in both terrestrial [7], [8], [9], [10], [11] and aquatic [12], [13], [14], [15] ecosystems.

More than 4500 non-native and invasive species live in natural ecosystems across the United States [5]. Non-indigenous species may harm native ecosystems through competition, predation, habitat modification, and hybridization with native species [3], [4], [5]. Invasive species are implicated as factors in listing ≥160 native species as threatened or endangered in the United States [5]. More generally, the overall environmental and economic impact of invasive species totals ∼US$137 billion annually in the United States, negatively affecting not just natural systems but also agriculture, aquaculture, forestry, transportation, utilities, recreation, and human health [5], [16].

The process of a species' invasion of a new area generally occurs in several steps: introduction, establishment, and spread [17]. Establishment often involves a “lag” phase, lasting even many years, in which the species is present in relatively low numbers in a restricted geographic area [18]. During the final phase, the species expands rapidly until it reaches its maximum distribution potential in the new landscape. The “rule of tens,” introduced by Williamson [17], holds that of species introduced into a new area, only ∼10% become established, and of species that become established, only ∼10% spread successfully. However, a recent study of species introduced from Europe to North America and *vice versa* found that ∼50 percent became established and ∼50% of those were able to spread [19]. Recent niche modeling applications suggest that these ‘numbers’ rules are constrained closely by the suitability of the landscape being invaded for the species [20], although the absolute nature of these constraints has been debated [21], [22], [23].

Common carp (*Cyprinus carpio*), tench (*Tinca tinca*), grass carp (*Ctenopharyngodon idella*), and black carp (*Mylopharyngodon piceus*) represent respectively a long-introduced and well-established invasive species (common carp), a long-introduced but less-successful non-native species (tench), a relatively recent but successful invasive species (grass carp), and a species being used in U.S. aquaculture that has not as yet become established in natural ecosystems (black carp). Our objective was to develop niche models for each species based on its native range and then project the niche model rule sets onto North America to identify areas at risk for establishment. This would allow us to test both distributional predictions quantitatively with independent occurrence data.

We define the “ecological niche” of species as the set of abiotic parameters within which a species is able to maintain populations without immigrational subsidy [24]—this “scenopoetic niche” can be distinguished from niches more closely related to interactions among species (the “Eltonian niche;” [25]) principally by the spatial scale at which it is manifested. We used landscape-scale environmental variables based on climate and topography, and developed tests of stability of ecological niches across the time span of the invasion events [26]. Our ENM application takes advantage of the fact that native populations have had more time and opportunity to “explore” environmental space via dispersal and colonization, and thus are likely to be most informative regarding niche dimensions [27], although combinations of models based on native and introduced distributional areas may be optimal for prediction [28].

Because the geographic extent of a species' potential distributional area is independent of its success to date as an invader, depending rather on time and opportunity for access to the region being invaded [29], we hypothesized that common carp and tench occurrences would already have filled a substantial part of their potential ranges, given multiple introductions for over 150 yr in North America. Grass carp populations are expanding still [30], however, and have probably not yet reached their full geographic potential after almost 40 yr since introduction, whereas black carp are only recently imported into North America, and are not as-yet established in natural waters. We wished to determine whether niche models could detect and demonstrate this pattern where species expand into new environments, out to the bounds imposed by their scenopoetic niches [29], [31], [32]. Finally, to the extent that potential ranges *can* be reconstructed predictively and robustly we wished to determine what geographic “behavior” we might expect from the newest arrival, the black carp?

### Reactive vs. proactive approaches

Invasive species remediation efforts have too-often been highly reactive in nature [1], which means that species tend to establish populations before their threat is recognized. ENM allows assessment of potential geographic areas at risk for invasion before the introduction even takes place [33], [34], [35]. One method of generating niche models, the evolutionary computing algorithm GARP, uses a variety of rule-building methods in an iterative machine-learning process to generate rule sets [36]. Environmental data sets in the form of raster grids are entered into GARP, along with georeferenced occurrence points from the species' native range. By comparing environmental parameter combinations of known occurrence points against those of points randomly sampled from areas from which the species is not known to occur, GARP “learns” a pattern of relationships in the form of a rule set. Rules are iteratively combined, altered, and refined to maximize accuracy. The final result of the algorithm is the niche model, which describes a species' niche as a multi-dimensional hypervolume in ecological space [37], [38].

Although niche models are generated using environmental data and occurrence data from the species' native range, the rules contained within models are defined in environmental terms only, and are independent of geographic area. Hence, rule sets can be applied in any geographic area to identify areas of potential occurrence. We selected GARP as a modeling implement because distributional predictions derived from GARP models have proven accurate under a variety of circumstances [39], and because GARP is robust to small occurrence data sets [40]. Machine-learning techniques, such as GARP, are powerful because the modeling of non-linear functions with large numbers of variables becomes feasible as compared to traditional statistical approaches [41]. Although GARP was not ranked particularly highly in some broad intermodel comparisons [42], these comparisons have been demonstrated to be largely artifactual in more recent analyses [43].

### The fishes

Common carp, native to Eurasia, were first introduced in North America in 1831 [44] and were intentionally released throughout most of the United States by the U.S. Fish Commission in 1877–1898 [45], [46]. Now considered an invasive nuisance, common carp are established in every U.S. state except Alaska [47], and seem to have established almost completely across their potential distributional range [15]. The impact of common carp on native aquatic species has been primarily through habitat modification, as it stirs up substrates, uprooting plants and muddying the water [46], [48]. Common carp occupy many microenvironments, are highly fecund, and spawn in shallow, slow-flowing water [49].

The U.S. Fish Commission originally imported tench into the United States in 1877 [45], and eventually provided tench stocks to 36 U.S. states [50]. However, many of the introductions seem to have been unsuccessful, possibly due to biotic interactions [47], [50]. Tench populations are probably established in California, Colorado, Connecticut, Idaho, Washington, Oregon, New Mexico, Maryland, New York (U.S.), and British Columbia (Canada), but their current status in many of these areas is uncertain [47], [51]. Tench usually spawn in weedy shallow areas, and their diet consists chiefly of insects and mollusks [52].

Grass carp were imported into the United States from Malaysia and Taiwan for aquaculture in 1963, and were released into natural waters shortly thereafter in Arkansas [53]. By 1993, grass carp were established in Arkansas, Kentucky, Illinois, Louisiana, Missouri, Mississippi, Tennessee, and Texas [30]; evidence of reproduction has been recorded from the Mississippi River drainage of all of these states except Texas [47]. Grass carp have already adversely affected U.S. ecosystems in several areas [54]. In China, grass carp generally prefer large rivers and lakes, requiring long rivers (50–180 km) with sufficient discharge (>400 m^3^/sec) and velocity (>0.8 m/sec) for successful reproduction [55], and eat mainly submerged vegetation [53].

Black carp have been used in U.S. fish farms as biological control against snails, which are secondary hosts for parasites that infect commercial fish [56], [57]. Although concerns about black carp escape, establishment, and potential impacts on North American ecosystems have been raised [58], the aquaculture industry maintains that use of the species is necessary [59]. Use of black carp has been focused in southeastern U.S. fish farms that raise channel catfish (*Ictalurus punctatus*) and striped/hybrid bass (*Morone* sp.) [60]. Although no black carp breeding populations are as yet known in the U.S., ∼30 black carp escaped into the Osage River from a fish farm in Missouri in 1994 [47]. Recently, black carp have been captured in Illinois [61] and Louisiana [51]; it is not known whether self-sustaining populations are established. In their native range, black carp inhabit major rivers, large tributaries, and lakes; they are river breeders that require a swift current for successful development, and spawning habitat has been described as similar to that of grass carp [60].

## Methods

We constructed niche models using 58 native range occurrence points for common carp, 292 for tench, 41 for black carp, and 38 for grass carp. We compiled these data from specimen records in museum collections via online databases [62], [63], verified records from Chinese museum collections, and species accounts in scientific literature [64], [65], [66], [67]. We inspected fish collections from the Chinese museums and identified a random subsample of approximately 10 specimens per species to assure correct species assignments. In all cases, we excluded all points outside the known native range from the model-building (training) data pool. We are interested in the potential for ENM as a tool in assessing invasion threat *before introduction*; therefore, points from the invaded range were deliberately excluded from the model-building process for all species. For each species, we used all available unique, verified native range occurrence points; consequently, sample size varied among species. GARP predictions have been shown to reach 90% of maximum accuracy using only 10 training points, with smaller incremental changes in accuracy when more data are included, such that most of the change in accuracy occurs below 20 data points [68]. As all our data sets exceed 20 points, we expect the effect of variable sample size on model accuracy to be negligible.

For North American occurrences, we obtained 1303 points for common carp, 30 for tench, and 47 for grass carp through the above online databases and the USGS Nonindigenous Aquatic Species database [51] from records of fish not directly stocked (black carp are not as yet known from natural waters). For records not already georeferenced, we assigned latitude and longitude coordinates with the U.S. National Geospatial-Intelligence Agency GEOnet Names Server [69], the USGS Geographic Names Information System [70], and detailed printed map resources [71].

The native range of black carp includes the major Pacific drainages of eastern Asia from about 22°N to 51°N latitude, including areas in China and Russia [72]. Grass carp have a similar native range: the Pacific slope of Asia from the Amur River Basin to the West River, including areas in China, Russia, and Northern Indochina [53]. The native range for tench includes Europe and parts of western Asia [52], [64]. Because native range limits for common carp are poorly understood, as the species occurs throughout Eurasia, having been spread and released by humans there for centuries [47], we treated occurrences throughout Eurasia as native range for this species (after previous treatment of this species [15]). Any questionable occurrence points (e.g. market-collected specimens) were removed from the native range data pool. Duplicate occurrence points were also removed, leaving only verified, unique occurrence points.

Environmental data consisted of 15 layers (“coverages”) summarizing aspects of the ecological landscape of both the native and introduced ranges (Table 1). We were limited to only those environmental variables for which data is available. While we do not consider that the available coverages represent all possible variables affecting species distributions, we included all coverages in model-building that might either directly affect species occurrence, or act as proxies for unavailable data. Data layers varied in spatial resolution (the size of individual cells or pixels in a given grid layer), consequently, layers were resampled to 0.01×0.01° prior to analysis. We resampled layers for the common carp native range only to 0.05×0.05° resolution because of the vast area of analysis and associated computational restraints. Although higher spatial resolution is desirable, range predictions using GARP and similar ENM tools are only modestly affected by changes in resolution [73], [74].

**Table 1:** Environmental data layers used in the development of the models presented herein.

Description	Source	Resolution	Excluded Coverages			
			Common carp	Tench	Grass carp	Black carp
Diurnal temperature range	IPCC	0.5° lat-long				
Ground frost frequency	IPCC	0.5° lat-long		x		
Maximum temperature	IPCC	0.5° lat-long				x
Mean temperature	IPCC	0.5° lat-long		x		
Minimum temperature	IPCC	0.5° lat-long			x	x
Precipitation	IPCC	0.5° lat-long				
Solar radiation	IPCC	0.5° lat-long		x		
Vapor pressure	IPCC	0.5° lat-long		x		
Wet day frequency	IPCC	0.5° lat-long				
Percentage tree cover	UM	0.5 km				
Aspect	USGS	1.0 km		x		
Elevation	USGS	1.0 km				
Flow accumulation	USGS	1.0 km				
Slope	USGS	1.0 km		x		
Topographic index	USGS	1.0 km				

IPCC: Intergovernmental Panel on Climate Change, Climate Data Archive [101]. UM: University of Maryland [102]. USGS: United States Geological Survey, HYDRO1k Elevation Derivative Database [103].

Prior to generating niche models, we evaluated the environmental coverages with a jackknife process, an analysis designed to maximize predictive accuracy while culling coverages prone to spurious overfitting [14], [75], [76]. This procedure allowed us to optimize environmental layer inputs for each niche model in terms of minimizing model omission error (i.e., exclusion of independent test data points from model prediction) [77]. As such, for each species, we built models based on all combinations of *n*−1 data layers, where *n* is the total number of layers. Occurrence data were randomly divided into training and test sets (50% each); 20 models for each *n*−1 layer subset were built and tested using these training and testing subsets. We calculated correlations between inclusion of each and omission error in the test data set, and removed layers showing positive correlations with omission error (*r*>0.1) from subsequent analyses. We repeated the jackknife procedure with the reduced set of data layers; when no strongly positive correlations with omission remained, the remaining subset of data layers was designated for use in the final model building process.

For generating final models, occurrence points were divided randomly into training and validation subsets. For all models (except tench; see below), 20 native-range occurrence points were excluded from model building and reserved for independent model-set validation. For tench models, because of the larger data set available for tench native range, we used 20% of the data (58 points) for independent validation. The GARP program further divides training data into intrinsic training and extrinsic testing subsets (80% and 20% respectively herein) prior to each model building process [78]. All experiments were performed using the desktop version of GARP [79].

Models were generated until 20 models with 0% extrinsic omission were compiled (i.e., all of the extrinsic testing subset completely predicted), and remaining models were discarded. We calculated the median of the commission index (calculated as the proportional area predicted present [80]) across all zero-omission models, and selected the 10 models with the lowest deviation from the median as the best model-set used in the distributional prediction [78]. We used the pixel-by-pixel sum of these 10 models (projected across both native ranges and North America), as inclusion of more models has not increased model accuracy or interpretability in limited experimentation.

Validation points set aside prior to model building were then overlaid as an independent test of model-set accuracy. We calculated percent omission (“%O”) as 1 minus the weighted proportion of validation points predicted by 0–10 of the best model set. For example, if 17 of 20 validation points were correctly predicted by all 10 of the best models, 2 by 8 and 1 by 6, then %O = 1−((17+1.6+.6)/20) = 4%.

We evaluated model-set sensitivity and specificity using the Area Under the Curve [AUC] in a Receiver Operating Characteristic [ROC] analysis [81], [82], [83]. In ROC, each pixel in the landscape receives scores from the diagnostic test being evaluated, in this case based on the predictions for the independent testing data. These data are then graphed on a sensitivity vs. 1-specificity plot (sensitivity and specificity determined using a standard 2×2 confusion matrix, with absence information based on all sites from which the species has not been detected previously), and the area under the curve is calculated. This AUC is compared to a “line of no information” with a slope of 1 (AUC = 0.5). No difference between the two AUCs indicates that the best model set is predicting presence no better than at random [82]. The AUC can be interpreted as the probability of a model set correctly predicting presence in a randomly selected grid cell. Because we used a conservative estimate of commission error, even a perfectly accurate model cannot achieve a perfect AUC of 1.0, and AUC scores will be lower for equally accurate model sets as the percent of the total study area predicted by the model set increases [84]. We are aware of the problems inherent in AUCs and their interpretation [43], [85], and interpret our results cautiously as a result.

We calculated the AUC for each model set and tested it against AUC = 0.5 using a *z*-test to determine significance [81]. We also calculated the maximum AUC (AUC_max_) possible for each validation data set, given the distribution of validation datapoints available. We take the difference between AUC and AUC_max_ as a more informative measure of model set accuracy than the AUC alone [84]. To account for differences in AUC scores, we also calculated percent of the total study area predicted by all 10 best-subsets models.

After analysis on the native range, the best models for each taxon were projected onto central North America (∼25–54°N). We overlaid data points documenting non-native populations on these model predictions. We analyzed predictions with ROC analysis, as above, with the non-native occurrences as independent validation data. We also created maps of weighted proportion of area predicted present within hydrologic units for the lower 48 states by intersecting niche model grids with USGS 6-digit HUC polygons [86].

## Results

**Figure 1:**
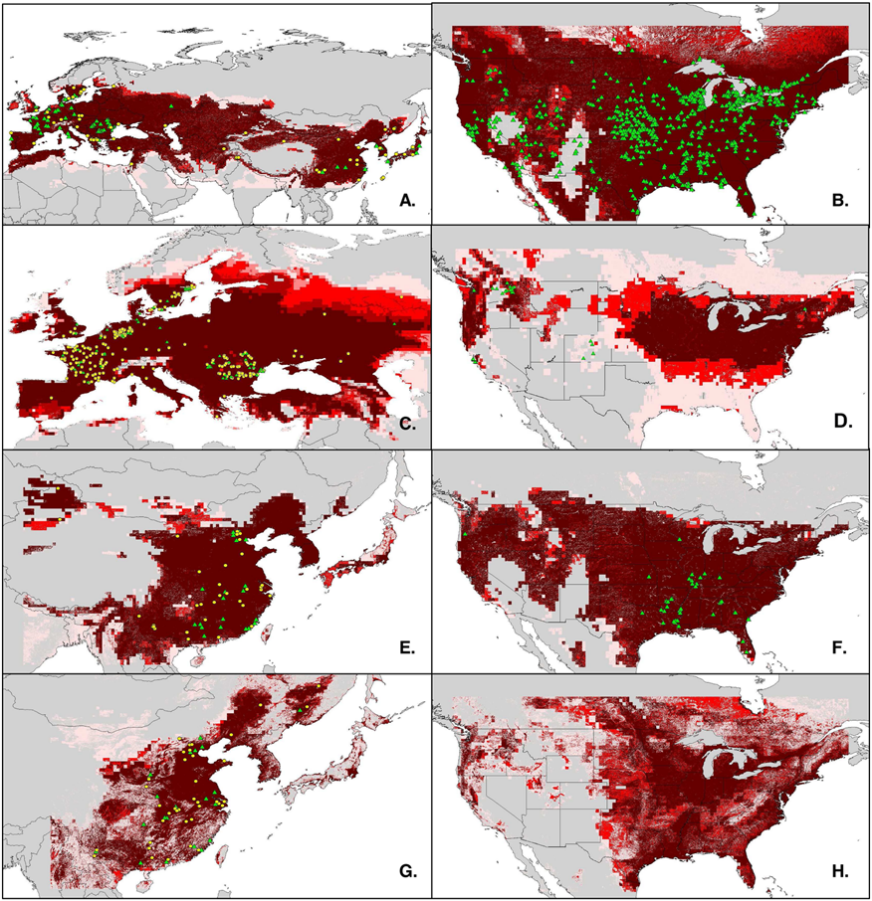
Niche models in native ranges and in the United States. Ecological niche models for common carp (A, B), tench (C, D), grass carp (E, F), and black carp (G, H) on native and U.S. landscapes. Shading indicates the predicted suitability predicted (brick red = 7–10 models, canary red = 4–6 models, pink = 1–3 models). Occurrence points for each species are shown as training data (yellow circles) in the species' native range (A, C, E, G) or independent validation data (green triangles) in the native or introduced ranges (B, D, F, H).

The best suite of coverages, as determined by the jackknife procedure, for each species is shown in Table 1. All AUCs for all taxa in both native and invasive model tests were significantly better than random (*P* = 0.01). Given this validation, we visualized both the native and non-native potential distributional areas for each taxon (Figure 1, Table 2), with details as follows. The common carp model predicted native presence across most of Eurasia; all 20 validation data points were predicted correctly by all of the best-subsets models (%O = 0, AUC = 0.80). For the non-native range, all 10 best models predicted a broad potential distribution covering much of the surface of the continental United States. Of 1303 occurrences available for common carp in North America, 991 were predicted present by all best-subsets models, with another 118 predicted present by 9 of the 10 best models; at the other end of the spectrum, 28 points were not predicted by any of the 10 models (%O = 8.8, AUC = 0.62). The native-range tench model performed well, with 10 models correctly predicting 57 of 58 validation points (%O = 0, AUC = 0.80); projection of this model to North America predicted presence across most of the upper Midwest and the eastern United States, and in parts of the northwest. Ten of the 30 North American tench validation points were predicted by all 10 models; 16 were predicted by 1–9 models; and five remained unpredicted (%O = 54.7, AUC = 0.62).

**Table 2:** Statistics describing the results of ecological niche model validations.

Niche model	AUC	SE	AUCmax	SE	% O	# trn. pts.	# vld. pts.
Common carp Native	0.8509*	0.0540	0.8509	0.0540	0.0	38	20
Common carp Intro.	0.6215*	0.0083	0.7315	0.0080	8.8	-	1303
Grass carp Native	0.8616*	0.0539	0.8616	0.0539	0.0	18	20
Grass carp Intro.	0.7078*	0.0427	0.8042	0.0386	19.8	-	47
Black carp Native	0.8745*	0.0506	0.9082	0.0445	6.5	21	20
Black carp Intro.	-	-	-	-	-	-	-
Tench Native	0.7955*	0.035	0.8006	0.035	0.0	234	58
Tench Intro.	0.6242*	0.0548	0.9498	0.0278	54.7	-	30

* p<0.001.

AUC: Area under the curve derived from ROC analysis. SE: Standard error of AUC. AUCmax: Maximum AUC, calculated with each independent validation data point receiving the maximum score. %O: Percent omission, a measure of model omission error across the 10 best model set. # train. pts: Number of training points for each niche model. # valid. pts.: Number of validation points for each niche model.

The grass carp model encompassed its known native range in eastern Asia. All 20 validation occurrence points were predicted present by all of the best models (%O = 0, AUC = 0.86). The projection to North America was more limited than for common carp (Table 2), but covered extensive portions of the eastern, central, and northwestern United States (Figure 1d). Of 47 grass carp testing points available from North America, 36 were predicted present by all models, and 2 were not predicted by any models (%O = 19.8, AUC = 0.71).

The area predicted for black carp is smaller, both on its native range and in North America (Table 2). Seventeen of the 20 native-range validation points were predicted by all models, and all points were predicted by ≥2 models (%O = 6.5, AUC = 0.87). Most of the eastern United States is predicted as suitable for black carp by these models (Figure 1f). Because black carp are not known to be established in North America, and the few records of this species [51], [61] are recent escapees, no independent validation points were available for black carp in North America.

## Discussion

The ecological niche models performed well in predicting independent testing data across native-range landscapes, including areas outside the actual native range. For example, black carp are predicted to find parts of northern Indochina and Japan suitable (Fig. 1): in the literature, we see that black carp have already become established in the Tone River system in Japan [72], [87] and probably in parts of Vietnam [88]. Although grass carp and black carp have similar native range limits, the distribution of occurrences within the range is dissimilar: as a consequence, grass carp predictions covered broader areas on both native and non-native landscapes.

Niche models predicted the known dimensions of the introduced range for common carp and grass carp successfully, in the former case in close agreement with the results of a previous study [15]. This result is significant, given that no information about the biology of these fishes or their non-native occurrences was included in the modeling effort. The models did fail to anticipate some of the introduced occurrence points and omission error was higher for the introduced-range tests, although only 2% of U.S. common carp points were not anticipated by the model predictions. Failures of ENM to fully predict an invaded range could occur when the invasive species is limited in its native range by biotic interactions and is released from those pressures in the invaded range, or when an island species has environmental tolerances that exceed the limits it encounters in its small native range [18], [34], [89]. Given the large native ranges of the carp species investigated here, and the common carp in particular, we do not consider either hypothesis a likely explanation in this case. Most of the unpredicted points for common carp occur in the southwestern U.S. in three major drainages (Colorado, Rio Grande, and Great Basin). All three of these systems have highly altered hydrology, which have been indicated as a primary reason for fish invasions [90], and as such aquatic environmental conditions there may not be representative, and may be dependent on smaller-scale interactions that cannot easily be captured by coarse-resolution environmental data used in this study.

The non-native grass carp model, although it performed statistically significantly better than random expectations, nonetheless had a 19.8% omission rate, owing to several points falling within small areas predicted by few or none of the best-subsets models. Upon closer examination, many grass carp testing points were outside (but close to) predicted areas by <1 km (Figure 1f), which is equal to or less than the native spatial resolution of the environmental data. Imprecision of coordinates for both native and non-native occurrence points may also have played some part in creating this elevated omission error.

The high omission of the introduced range projections of tench merits further discussion. One omitted point in Washington was slightly outside areas of high prediction, as were 3 points in California, so again data precision may come into the picture. Perhaps more importantly, though, tench have not established successfully in many areas predicted by the model where they have been introduced intensively. Baughman [50] found that states in the Great Lakes Region received 13,849 tench from the U.S. Fish Commission in 1886–1896 for introduction into various waters. The models predict potential distributional areas for tench across much of the Great Lakes region, but (other than a limited established population in the Great Chazy River in upstate New York) tench have failed to become established there [47], [51]. Kolar and Lodge [91] also predicted that tench would be successful in the Great Lakes region based on multivariate analysis of life-history characteristics, habitat needs, and invasion history. The reasons for the broad failure of tench introductions are currently not known, although some observational evidence suggests that biotic interactions with sunfishes (Centrarchidae), which are not native to Europe, may be responsible [50]. Another possibility is that environmental factors acting at resolutions finer than the data considered herein may be interacting negatively with the biology of the species. The disparity between our predictions and tench occurrences demonstrates the caution needed when interpreting these predictive models.

**Figure 2:**
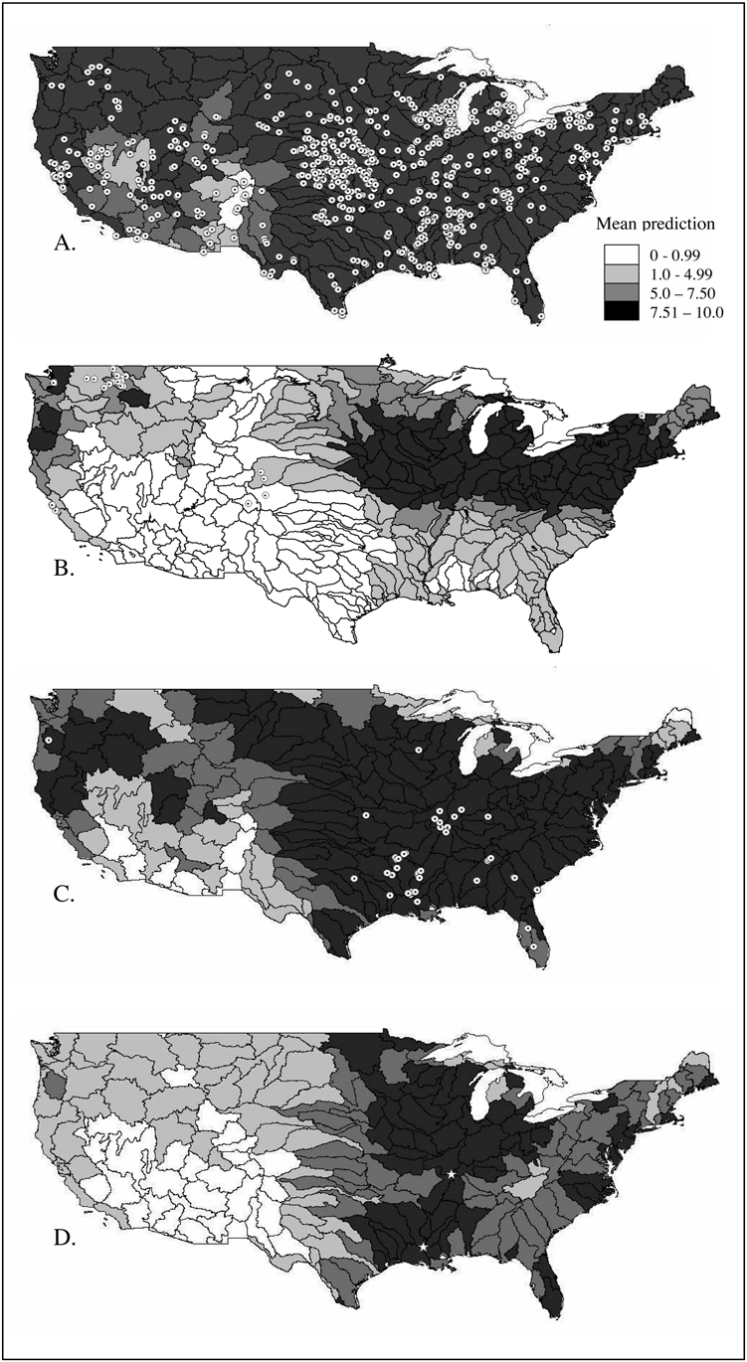
Invasive range predictions by USGS hydrologic unit. Mean ecological niche model predictions at the level of hydrologic unit (USGS 6-digit HUC) in the United States, for common carp (A), tench (B), grass carp (C), and black carp (D). Shade indicates mean suitability from the niche model outputs across all pixels within each HUC polygon (see legend). Known (testing) occurrence points are indicated by dotted circles. Recently captured black carp [23], [33] are shown with stars (D).

That several testing occurrence points fell slightly outside areas predicted at high levels for common carp, tench, and grass carp might suggest that models could be improved with higher-resolution environmental data and occurrence data. However, this improvement would not come easily, as the two data sets must be improved in tandem—the best resolution possible will be limited by the *coarser* of the two resolutions. In the meantime, it may be more appropriate to quantify risk at coarser resolutions. To this end, we evaluated mean model prediction across hydrologic units (USGS 6-digit HUC units) and compared them qualitatively to the non-native occurrence data (Figure 2). Evaluating predictions at the “pixel” scale may give a false picture of fine-scale accuracy. The grass carp niche model had 19.8% omission when evaluated at the pixel scale, but coincidence with HUCs in which there was high model agreement in prediction of presence was better (Figure 2C): evaluated at this resolution, the tench model identified the areas where tench managed to establish populations (Figure 2B). Moreover, the HUC-level maps avoid problems with spatial autocorrelation and the independence of testing points, and summarize the data at a more appropriate scale and provide a more interpretable map of risks for managers.

Although landscape-scale parameters are important in limiting fish distributions [92], exclusive use of this type of data can complicate potential distribution predictions for aquatic species, as we end up using putative proxies for instream parameters that are probably the truly causal variables. For example, while a comprehensive water temperature coverage is not presently available, air temperature interacts with factors, including dams, tree cover, volume, and groundwater input, to determine stream temperatures [93]. Given the limited data available, we included all layers that might provide useful surrogates, such as percent tree cover and solar radiation coverages as possible surrogates or partial surrogates for water temperature. Hence, ENMs could be improved by integration of datasets for aquatic parameters; in addition, several smaller-scale environmental and biological factors that influence occurrences of fish [94] could also be included. In the case of tench, biotic interactions may be limiting their establishment success: in cases like this one, ENMs can be viewed as the top layer of a multi-scale filtering framework, under which finer-scale models could improve predictions [95], [96].

Common carp have spread so broadly that their current range limits in North America reach (and in some areas exceed) their modeled potential distributional area [15]. In contrast, tench have been unsuccessful in establishing in many areas predicted as suitable by the ENMs; similar constraints could affect black carp, but anticipating their behavior in novel situations is not simple. More practically, resource managers may not be willing to take the risk that unforeseen biotic or environmental interactions may limit black carp establishment in areas predicted as suitable, given the possible negative impacts.

The negative ecological and economic impacts caused by establishment of non-native species in the United States are well known, but can be difficult to anticipate [1]. Black carp are molluscivores, and their presence may put native mollusks at risk, particularly in the southeastern U.S., which is home to 90% of the nation's threatened and endangered mollusk species [97]. Black carp can live for >15 years [88], and are capable of consuming large quantities of mollusks (four-year-old black carp consumed 1.4–1.8 kg of bivalves *per day* [98]!). Black carp ENMs predicted presence across much of the southeastern U.S., where many of the aquaculture facilities that use black carp are located [60]. Black carp have already escaped captivity in the United States–though not included in the present analyses, this species has been captured recently in Illinois [61] and Louisiana [51], both in hydrological units predicted as high risk (Figure 2D). The niche models predict presence in several watersheds containing major rivers, including almost the entire course of the Mississippi River, which appear to meet the hydrological requirements of black carp reproduction (Fig. 2d).

ENM provides the opportunity to assess invasion potential proactively by using occurrence data from museum collections and the scientific literature. Under Section 1105 of the pending National Aquatic Invasive Species Act of 2007 (NAISA) [99], U.S. federal agencies would have to complete a screening process for planned importations of live aquatic organisms, and make a determination to allow or restrict importation within 180 days of receiving a request for permission to import aquatic organisms [100]. If federal agencies adopted predictive ecological niche models, detailed maps of hydrologic units at risk, combined with brief synopses of the biology of the “new” species, could be distributed quickly to regional or state managers. Scientists with expert knowledge of local ecosystems could then evaluate risk of establishment and invasion by interpreting niche models and the natural history of the species.

Although not all species have similar (broad) invasion potential, our common carp analyses demonstrate that some species will eventually expand their ranges to approximately match the extents that we predicted. We also demonstrated potential range expansion in the more recently introduced grass carp. Tench occurrences, however, did not fill much of the geographic range predicted by the niche model, illustrating the difficulty in predicting the result of introductions given the multitude of factors that can determine the outcome. We believe, like Nico et al. [60], that black carp present a serious invasion threat: for this situation, we have identified regions at highest risk. While it is not possible to anticipate whether introduced species, like black carp, will exhibit the invasive potential of common carp *versus* the relative invasive ineptness of tench. However, regulatory agencies should give careful consideration to ecological niche models as an integral tool in achieving an effective strategy to limit potential negative impacts by invasive species.
